# The efficacy and safety of Lianhua Qingwen (LHQW) for coronavirus disease 2019 (COVID-19): A protocol for systematic review and meta analysis

**DOI:** 10.1097/MD.0000000000020979

**Published:** 2020-07-24

**Authors:** Qiongshuai Zhang, Fang Cao, Guangcheng Ji, Xiaohong Xu, Yihan Sun, Jiannan Li, Xun Qi, Shaoqian Sun, Yufeng Wang, Bailin Song

**Affiliations:** a Department of Acupuncture and Tuina, Changchun University of Chinese Medicine, Changchun; b Department of Acupuncture, The First affiliated Hospital of Henan University of TCM, Zhengzhou; c Department of Rehabilitation, The Third Affiliated Hospital of Changchun University of Chinese Medicine; d Graduate school, Changchun University of Chinese Medicine; e Department of TCM, Changchun University of Chinese Medicine; f Department of Rehabilitation, Chian-Japan Union Hospital of Jilin University; g Department of Tuina, Traditional Chinese Medicine Hospital of Jilin Province, Changchun, China.

**Keywords:** coronavirus disease 2019, COVID-19, Lianhua Qingwen, meta-analysis

## Abstract

**Background:**

Since the outbreak of Novel Coronavirus Pneumonia (NCP), it has swept the world with rapid development. Up to now, there is no effective drug to treat it. Lianhua Qingwen has been used in the treatment of COVID-19 in China, but there is no systematic review about it. This study will systematically evaluate its efficacy and safety in the treatment of COVID-19.

**Methods:**

We will search electronic database of PubMed, EMBASE, Cochrane library, Web of Science (WOS), China National Knowledge Infrastructure (CNKI), Chinese Biomedical Literature Database (CBM), Chinese Scientific and Journal Database (VIP) and Wan Fang database (Wanfang) for the literature of RCTs of Lianhua Qingwen capsule for coronavirus disease 2019 (COVID-19). We will also search the Chinese Clinical Trial Registry (ChiCTR) and ClinicalTrials.gov (www.ClinicalTrials.gov) for ongoing trials with unpublished data, and the Conference abstracts will be searched manually. We will use the Cochrane Handbook for Systematic Reviews of Interventions to assess the risk of bias. The protocol will be conducted according to the approach and Preferred Reporting Items for Systematic Review and Meta-Analysis Protocols (PRISMA-P).

**Results:**

The study results will provide evidence of the efficacy and safety of Lianhua Qingwen (LHQW) for coronavirus disease 2019 (COVID-19).

**Conclusion:**

The result of the study will be published in a peer-reviewed journal.

**PROSPERO registration number:**

CRD42020180877.

## 1 Introduction

Novel coronavirus pneumonia (SARS-CoV-2) was found in Wuhan, China, in early December 2019,^[1]^ showing a high contagious and rapid transmission characteristic, which could cause severe new crown pneumonia (COVID-19)^[2,3]^ in a certain proportion of patients. According to the WHO report,^[4]^ about 13.8% of the patients are severe patients, 6.1% of them are critical patients, and some of them may develop into critical patients due to poor or delayed treatment. The clinical manifestations^[5,6]^ of COVID-19 are fever, dry cough, dyspnea, muscle or joint pain, diarrhea, and pneumonia, which can lead to respiratory failure or even death in severe cases, COVID-19 is causing a global pandemic.^[7,8]^ Through comprehensive prevention and control, Chinas epidemic has been gradually under control, but at present, CONVID-19 is spreading rapidly outside China. Globally, as of 10:44 pm CEST, May 21, 2020, there have been 4,904,413 confirmed cases of COVID-19, including 323,412 deaths, reported to WHO.^[9]^ Which has become a huge challenge for global public health.^[10–12]^

Lianhua Qingwen is mainly made of forsythia, honeysuckle, isatis root, menthol, liquorice, agastache, almond, gypsum, ephedra, Rhodiola, houttuynia and other Chinese herbal medicines,^[13,14]^ and it has been used for fighting against the epidemic in China,^[15,16]^ So far, there have been some relevant clinical reports^[17,18]^ on COVID-19 treated with Lianhua Qingwen. Therefore, this study will collect the relevant research, and then review the effectiveness and safety of Lianhua Qingwen for COVID-19.

## 2 Methods

Preferred reporting items of systematic reviews a meta-analysis protocol (PRISMA-P) 2015^[19]^ will be used as a guideline in performing of the systematic review.

### 2.1 Inclusion criteria

#### 2.1.1 Types of studies

We will include randomized controlled trials (RCTs) of Lianhua Qingwen for COVID-19 in the treatment groups. There will be no language restrictions

#### 2.1.2 Types of participants

Patients diagnosed with COVID-19 of any age, gender, and the racial group will be included.

#### 2.1.3 Types of interventions

The treatment group with Lianhua Qingwen Capsule or Lianhua Qingwen Granule for COVID-19 and the control group may receive external treatment, placebo, no intervention or other pharmacological intervention will be included.

#### 2.1.4 Types of outcome measures

Primary outcome: severe type conversion rate; secondary outcome: the proportion of participants with fever, Proportion of participants with 1 or more adverse events, Health-related quality of life.

### 2.2 Search strategies

Electronic databases of PubMed, EMBASE, Cochrane library, Web of Science(WOS), China National Knowledge Infrastructure (CNKI), Chinese Biomedical Literature Database (CBM), Chinese Scientific and Journal Database (VIP), and Wan Fang database (Wanfang) will be searched to identify literature of RCTs of Lianhua Qingwen for coronavirus disease 2019 (COVID-19). Meanwhile, we will search the Chinese Clinical Trial Registry (ChiCTR) and ClinicalTrials.gov (www.ClinicalTrials.gov) for ongoing trials with unpublished data, and the Conference abstracts will be searched manually. We will search the above databases from inception to 30 Jun 2020 with no language restrictions.

The search strategy of PubMed is listed in Table 1. We will appropriately adjust the search strategy for different databases accordingly.

**Table 1:**
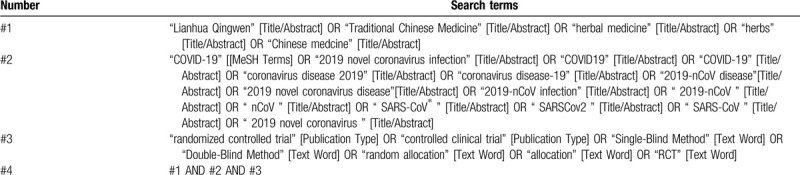
Search strategy for PubMed.

### 2.3 Data collections and analysis

#### 2.3.1 Selection of literature

**Figure 1:**
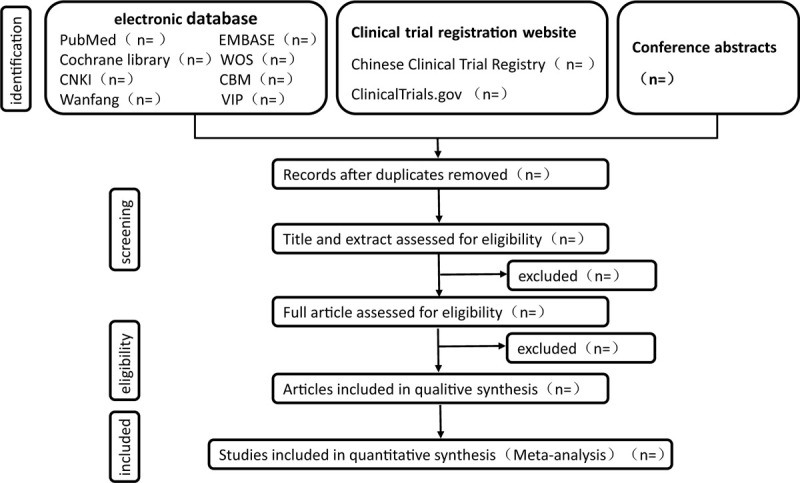
The screening process.

Two reviewers (CF and XQ) will identify the eligibility of the studies independently using inclusion and exclusion criteria by Endnote software (V.x9.0) to eliminating the duplicates, then they will exclude other non RCTs, such as animal experiment, case report or systematic review by reading titles and abstracts. Finally, they will read full papers of the screened articles to decide whether it could be included. If there is any dispute during this period, the 2 reviewers may refer to the third expects (BLS), they would make the decision by voting. All screening and managing processes of the articles will be performed with Endnote software. The screening process of this study will be carried out according to Figure 1

#### 2.3.2 Data extraction and management

Then 2 reviewers (YHS and JNL) will extract title, the first author, publication year, country, language, journal source; information of participants: gender, age, study design, sample size, intervention, type of measures, risk of bias assessment, and findings from include studies with Excel file.

The results will be cross-checked by the 2 reviewers, and any disagreements will be resolved by consensus, with any ongoing differences in opinion being arbitrated by a third reviewer (JNL).

#### 2.3.3 Assessment of risk of bias in included studies

Two independent reviewers (QSZ and FC) will evaluate the quality of the included trials by assessing the risk of bias by using the Cochran Collaboration Network Bias Risk Assessment Tool. The 2 authors will assess the risk of bias of sequence generation, allocation concealment, blinding of participants personnel and outcome assessment, incomplete outcome data, selective outcome reporting, and other bias. If there is disagreement during the assessing process, discuss with the third experts (YFW) to make decisions. The evaluation grades are low, high, and unclear risk of bias.

### 2.4 Dealing with missing data

If data of the included studies missed, we would contact the author for help. If it does not work, we will turn to follow strategies to evaluate the potential influence of missing data.^[20]^

1. Worst-case scenario analysis: All participants with missing data counted as failures.
2. Extreme worst-case/best-case scenario analysis: Participants with missing outcome data in the exercise arm counted as failures and in the control arm as success and vice versa.

### 2.5 Assessment of heterogeneity

Standard *I*^2^ test will be used for assessing the statistical heterogeneity, the significance level of *I*^2^ ≥ 50% indicates significant, otherwise, it means insignificant.

### 2.6 Assessment of reporting bias

If more than 10 studies are included, a funnel plot will be used to assess reporting bias, and if less than 10studies are included, *P* value will be used.

### 2.7 Data synthesis

Meta-analyses will be conducted when at least 2 studies included. We will conduct Statistical analyses using the RevMan software (version 5.3.5) Risk ratio (RR) with 95% CIs will be used to investigate dichotomous data. Standard mean difference (SMD) with 95% CIs or a weighted mean difference (WMD) will be used to analyze continuous data. The WMD will be used for the same scale or the same assessment instrument; SMD will be used for different assessment tools. The fixed-effect model will be utilized if the heterogeneity test indicates that there is no significant heterogeneity (*I*^2^ < 50%; *P* > .1); otherwise, the random-effects model will be used.

### 2.8 Subgroup analysis and investigation of heterogeneity

Subgroup analysis will be performed to determine the potential heterogeneity and inconsistency clinically and statistically, if enough data are extracted. Subgroup analysis based on: gender, age, whether patients in the included trials have basic diseases or not

### 2.9 Sensitivity analysis

We will conduct sensitivity analysis to assess the robustness and reliability of the pooled results. If the results are unstable, of high-risk bias studies may be excluded, otherwise, we will check the processing method of missing data (Worst-case scenario analysis: All participants with missing data counted as failures; Extreme worst-case/best-case scenario analysis: Participants with missing outcome data)

### 2.10 Grading the quality of evidence

We will make use of GRADE to assess the quality of evidence.

### 2.11 Ethics and dissemination

Ethical approval is not necessary for this study, for there is no information related to the individual patient. The systematic review will be conducted according to PRISMA guidelines, and we will show the assessment of the effect and safety of LHQW for COVID-19, and we will publish it in a peer-reviewed journal.

## 3 Discussion

LHQW Formula is a traditional Chinese herbal medicine prescription, and it is used for Influenza in China before, which also shows good clinical efficacy in treating Novel Coronavirus Pneumonia (NCP) resulted from SARS-CoV-2. LHQW was also composed into the Diagnosis and Treatment Programs of 2019 New Coronavirus Pneumonia (from fourth to seventh editions) formulated by the National Health Commission of China. Aiming to prevent and treat viral influenza.^[21]^ On April 12, 2020, the National Medical Products Administration of China issued that: the functional indications item of Lianhua Qingwen Capsule and Lianhua Qingwen Granule added “In the treatment of Novel Coronavirus Pneumonia, it can be used for fever, cough, and fatigue caused by light and common types”^[22]^ So far there is still no systematic review and meta-analysis on efficiency and safety of LHQW for COVID-19. We conduct this study, aim to provide evidence and guide for clinical decision making. We plan to publish this review within 2 months since the protocol published, then we will update it every 3 years.

## Author contributions

QSZ and BLS conceived and designed the protocol, QSZ, FC, and YHS registered the protocol review in the Prospero database and drafted the manuscript. YFW and XHX designed the search strategy. QSZ and XQ draft the protocol, QSZ, FC, YFW, YHS, XHX, JNL, XQ, SQS, GCJ, and BLS contribute to and approved the final manuscript of the protocol review.

**Conceptualization:** Qiongshuai Zhang, Bailin Song

**Data curation:** Guangcheng Ji, Xun Qi, and Jiannan Li.

**Funding acquisition:** Bailin Song

**Methodology:** Yihan Sun and Shaoqian Sun.

**Original draft:** Qiongshuai Zhang and Xun Qi.

**Project administration:** Yufeng Wang.

**Review & editing:** Yufeng Wang and. Bailin Song

**Software:** Shaoqian Sun, Jiannan Li
